# Insights into Systems for Iron-Sulfur Cluster Biosynthesis in Acidophilic Microorganisms

**DOI:** 10.4014/jmb.2206.06045

**Published:** 2022-08-19

**Authors:** Pérez Myriam, Paillavil Braulio, Rivera-Araya Javiera, Muñoz-Villagrán Claudia, Orellana Omar, Chávez Renato, Levicán Gloria

**Affiliations:** 1 Universidad de Santiago de Chile (USACH), Facultad de Química y Biología, Departamento de Biología. Av. Libertador Bernardo O´Higgins 3363, Estación Central, Santiago 9170022, Chile; 2 Universidad de Chile, Facultad de Medicina, Instituto de Ciencias Biomédicas, Laboratorio de Biología Molecular Bacteriana City, 8380453, Chile

**Keywords:** Fe-S cluster, Isc, Suf, acidophiles, oxidative stress, iron starvation, *Leptospirillum*

## Abstract

Fe-S clusters are versatile and essential cofactors that participate in multiple and fundamental biological processes. In *Escherichia coli*, the biogenesis of these cofactors requires either the housekeeping Isc pathway, or the stress-induced Suf pathway which plays a general role under conditions of oxidative stress or iron limitation. In the present work, the Fe-S cluster assembly Isc and Suf systems of acidophilic Bacteria and Archaea, which thrive in highly oxidative environments, were studied. This analysis revealed that acidophilic microorganisms have a complete set of genes encoding for a single system (either Suf or Isc). In acidophilic Proteobacteria and Nitrospirae, a complete set of *isc* genes (*iscRSUAX-hscBA-fdx*), but not genes coding for the Suf system, was detected. The activity of the Isc system was studied in *Leptospirillum* sp. CF-1 (Nitrospirae). RT-PCR experiments showed that eight candidate genes were co-transcribed and conform the *isc* operon in this strain. Additionally, RT-qPCR assays showed that the expression of the *iscS* gene was significantly up-regulated in cells exposed to oxidative stress imposed by 260 mM Fe_2_(SO_4_)_3_ for 1 h or iron starvation for 3 h. The activity of cysteine desulfurase (IscS) in CF-1 cell extracts was also up-regulated under such conditions. Thus, the Isc system from *Leptospirillum* sp. CF-1 seems to play an active role in stressful environments. These results contribute to a better understanding of the distribution and role of Fe-S cluster protein biogenesis systems in organisms that thrive in extreme environmental conditions.

## Introduction

Iron-sulfur (Fe-S) clusters are inorganic cofactors of proteins that play important roles in cellular processes, such as electron transport, catalysis, respiration, RNA modification and oxidation-reduction reactions in prokaryotic and eukaryotic cells [1]. These clusters are constituted of iron ions (Fe^2+^ or Fe^3+^ depending on structure type) and sulfur (S^2-^), where iron ions are coordinated with cysteine residues in the active center of the proteins. The structure of Fe-S clusters is named according to the disposition of these ions, and the most common are those with rhombic [2Fe-2S], and cubane [4Fe-4S] geometries[2, 3]. The main functions associated with Fe-S clusters are electron transfer (*e.g.*, ferredoxins) involved in the catalysis of redox reactions (*e.g.*, respiratory complexes), and in the sensing iron levels and oxidative conditions [4-6].

The structural versatility and chemical reactivity of Fe-S clusters mean that they have been incorporated as cofactors into many proteins. However, under oxidative stress, it is well known that reactive oxygen species (ROS) can damage these clusters, since they are the preferred target for superoxide ions (O_2_·^-^) and hydrogen peroxide (H_2_O_2_). Superoxide tends to be electrostatically attracted to the catalytic iron atom and the oxidation of this ion can destabilize the metal center, rendering the enzyme inactive and interrupting biological processes [7]. Hydrogen peroxide reacts with iron from the centers, triggering Fenton chemistry that generates hydroxyl radicals (OH·), which are highly toxic due to their ability to damage DNA [7]. Remarkably, ROS-induced iron release from these clusters further exacerbates oxidative stress in the affected cells.

In *E. coli*, two systems for the biosynthesis of Fe-S centers have been characterized, denominated Isc (iron sulfur clusters) and Suf (sulfur formation). Genes for Isc and Suf systems are arranged in the operons *iscRSUA-hscBA-fdx* and *sufABCDSE*, respectively [8, 9]. Isc is the housekeeping machinery that provides Fe-S clusters for proteins that participate in numerous cellular processes [2]. Conversely, the Suf system is active only under oxidative stress, and iron limitation [10]. In *E. coli* the transcription factor IscR, that contains a [2Fe-2S] (holo-protein), represses the *isc* operon and therefore its own expression. On the other hand, apo-IscR, together with OxyR and integration host factor (IHF), activate the Suf operon under stress conditions [11, 12]. IscR binds at three sites in the promoter of the *suf* operon. One of those sites overlaps with the binding site of the iron-dependent Fe^2+^-Fur repressor, which under iron starvation can lose its Fe^2+^, releasing the *suf* promotor region, and thus allowing IscR to bind and activate the expression of the complete *suf* operon [13].

Assembly of Fe-S clusters is carried out on scaffold proteins. The Isc system contains a cysteine desulfurase (IscS) that releases sulfur from L-cysteine to form a persulfide bond with one cysteinyl residue of the protein, which is transferred to the scaffold proteins IscU and IscA. Iron ions are obtained for the synthesis from CyaY protein and another unknown source [14]. Once assembled, Fe-S clusters are transferred from IscU to the apoprotein by the activity of HscA and HscB chaperone proteins [14, 15]. On the other hand, the Suf system consists of a cysteine desulfurase (SufS) which has the same function as IscS. However, IscS forms a complex with the helper protein (SufE) [8]. The source of iron is unknown for the Suf system. Synthesis *de novo* of Fe-S occurs on the scaffold proteins SufU and SufA. Then, the cluster is transferred to SufB, which forms a stable complex with SufC and SufD, where SufC is an ATPase involved in the dissociation of Fe-S clusters from SufB to transfer them to apoproteins [16]. Analysis of the phylogenetic distribution of these systems in prokaryotes has shown that Isc is limited to Proteobacteria, whereas Suf is likely to be the ancestral and most widespread system. The Suf system appears to be the sole system for Fe-S cluster biogenesis in archaea and cyanobacteria, and in many Gram-positive bacteria [17]. It is noticeable that thermophilic bacteria from the *Thermus* genus and radiation resistant bacteria from the *Deinococcus* genus have the stress-induced Suf system [18].

Extreme acidophilic bacteria are of great interest due to their capacity to live under harsh environmental conditions such acidic pH, high osmolarity and high heavy metal concentrations [19, 20]. These conditions contribute to creating highly oxidative environments that can induce damage to the biomolecules, thus affecting the architecture and integrity of the Fe-S clusters of proteins and likely the regulation of the corresponding biosynthesis pathways of these cofactors. Fe-S cluster-containing proteins dominate the proteome of the archaeon *Ferroplasma acidiphilum* [21]. They have also been reported in a number of acidophiles such as *Acidithiobacillus ferrooxidans*, and *Leptospirillum* sp. [22, 23]. Nevertheless, the abundance of these proteins in the proteome of iron-oxidizers and other acidophilic microorganisms remains unknown. In the same way, the strategies used by acidophilic microorganisms to satisfy the demand for Fe-S clusters remain poorly addressed.

In this work, by using a bioinformatic approach, we evaluated the Fe-S cluster biosynthetic pathways in a number of acidophilic microorganisms. In addition, we characterized the organization of the *isc* operon and its expression patterns under stress conditions in *Leptospirillum* sp. CF-1, a bacterium highly abundant in acidic bioleaching systems, and highly resistant to extreme oxidizing conditions.

## Materials and Methods

### Bioinformatic Prediction of Fe-S Proteins in Acidophiles

Genome sequences from iron- and/or sulfur-oxidizing acidophilic Bacteria (25) and Archaea (8) with complete or draft genomes available in the DDBJ/EMBL/GenBank databases were selected for the analysis. For comparison purposes, the sequences of twelve neutrophilic model bacteria (11) and archaea (1) were also considered. The selected genomes were uploaded to RAST (http://rast.nmpdr.org/) for prediction of Isc/Suf systems. The number of Fe-S proteins was predicted using Metal Predator (http://metalweb.cerm.unifi.it/tools/metalpredator/).

### Strains and Culture Conditions

*Leptospirillum* sp. CF-1 was grown in 9K BR medium (pH 1.6) containing 66 mM ferrous sulfate (Fe2SO4·7H2O) at 37°C with constant stirring at 180 ×*g* [24].

### Stress Induction

Oxidative stress induction was carried out as described previously [25]. Three liters of *Leptospirillum* sp. CF-1 culture were grown until the late exponential phase. Cells were harvested by centrifugation at 9,000 ×*g* for 20 min at 15°C. Then cells were washed once with acid water (10 mM H_2_SO_4_). Washed cells were resuspended in 100 ml of fresh medium and incubated for 30 min at 37°C. For oxidative stress induction, the cultures were supplemented with 260 mM Fe_2_(SO_4_)_3_ (ferric iron) as oxidative agent for 0.5 or 1 h at 37°C. Iron starvation was induced by reducing the concentration of ferrous iron as its energy source (FeSO_4_) to 33 or 0 mM for 3 h. Then, cells were collected by centrifugation, washed twice with acid water, and once with 10 mM sodium citrate pH 7.0, and stored at -80°C until further use.

### DNA Extraction

Genomic DNA purification was performed according to [26]. In brief, *Leptospirillum* sp. CF-1 was grown until the late exponential phase. Cells were harvested by centrifugation at 8,000 ×*g* for 15 min and washed twice with acid water (10 mM H_2_SO_4_) and once with 10 mM sodium citrate pH 7.0. Genomic DNA was isolated using the Wizard Genomic DNA Purification Kit (Promega, Cat_A1125, USA) according to the manufacturer’s instructions and stored at -20°C until further use.

### RNA Extraction and cDNA Synthesis

RNA was isolated using the RNeasy Mini Kit (Qiagen, Cat_74004, Germany). DNA was removed by DNase I treatment (Biolabs, New England) according to the manufacturer’s instructions. cDNA synthesis was carried out with the Affinity Script qPCR cDNA Synthesis kit (Agilent Technologies, Cat_600559, USA). The reaction mixture of 20 μl contained First Strand master mix, 0.1 μg/μl random primers, Affinity Script RT/RNase Block enzyme mixture, and 1 μg of RNA. The synthesis was carried out at 25°C for 5 min and then at 42°C for 15 min. The enzyme was inactivated at 65°C for 5 min and the cDNA was stored at -80°C.

### Determination of Co-Transcription of *isc* Genes

Co-transcription was studied by reverse transcription (RT)-PCR experiments. Primers for RT-PCR reactions (Table 1) were designed using the available gene sequences of *Leptospirillum* strain CF-1 [27]. The GoTaq DNA Polymerase kit (Promega) was used for PCR amplification according to the provider´s instructions, using the previously synthesized cDNA. The melting temperature of the primers (Tm) is indicated in Table 1.

### Determination of Relative Levels of RNA: Quantitative PCR Reaction

Primers of *Leptospirillum* sp. CF-1 designed for qPCR reactions are shown in Table 1. Quantitative PCR amplification was carried out with KAPA SYBR FAST qPCR kits (Kapa biosystems, Cat_KK4602) in accordance with the manufacturer’s instructions. The qPCR conditions were an initial denaturation at 95°C for 5 min, followed by 40 cycles of denaturation (95°C for 30 s), annealing (58°C for 20 s) and extension (72°C for 10 s). All these reactions were performed in a StepOne Real-Time PCR system (Applied Biosystems, USA). The relative abundance of each gene versus a constitutively expressed gene (*rrsB*: 16S rDNA) was determined. The results were expressed as means of three independent experiments.

### IscS Enzyme Activity

The activity of IscS in cell extracts was determined by measuring the production of thiocyanate from reaction mixtures containing cysteine, KCN, and protein extract, according to [28]. After 20 min at room temperature, 15%formaldehyde and 15% ferric nitrate were added, and the concentration of ferric thiocyanate was determined by absorbance at 460 nm. One unit of enzyme activity was defined as the amount of enzyme that catalyzes the formation of one nmol of thiocyanate/min; specific activities are given as units per µg of protein.

### Phylogenetic Analysis

The phylogenetic trees, using IscS and SufS protein sequences, were generated using the maximum-likelihood and JTT matrix-based model in the MEGA-X 10.1.8 software package [29]. The level of support for the phylogenies was gauged by 1000 bootstrap replicates.

### Statistical Analysis

Statistical analysis was performed using the one-way ANOVA test followed by Turkey’s test in GraphPad Prism 9. The differences were considered significant at *p* ≤ 0.05.

## Results

### Prediction of Fe-S Cluster Proteins Encoded in Genomes of Acidophiles

It has been reported that in *D. radiodurans*, a neutrophilic bacterium that can withstand severe oxidative stress, the content of Fe-S proteins is significantly lower than that of sensitive bacteria [30]. Therefore, we evaluated the number of Fe-S proteins encoded in the genome of acidophilic iron- and/or sulfur-oxidizing microorganisms. For this study, complete or draft genomes of 36 bacterial (25 acidophiles and 11 neutrophiles) and 9 archaeal (8 acidophiles and 1 neutrophile) strains were analysed by a genomic approach using the web server “Metal Predator” as described in Materials and Methods.

The analysis of data (Table S1) indicated that all the analysed strains of acidophiles have a number of genes coding for Fe-S cluster proteins with a range of 65-129 genes per genome in archaea and 73-145 in bacteria, with averages of 97.6 and 100.5, respectively (*p* > 0.05). Interestingly, the predicted number of genes for these microorganisms appears to be substantially lower than the number found in neutrophilic proteobacteria such as *E. coli* (154), *Salmonella enterica* (150), *Pseudomonas aeruginosa* (140), and *Klebsiella neumoniae* (137). In addition, according to our results, *D. radiodurans* and *Thermus aquaticus*, both of which are highly tolerant to oxidative conditions, possess 72 and 75 genes per genome, respectively. Similarly, other Gram-positive neutrophilic bacteria such as *Staphylococcus aureus* (49) and *Bacillus subtilis* (85) also harbour a reduced number of Fe-S proteins. The average numbers of genes that encode predicted Fe-S proteins in acidophilic archaea and bacteria suggest a slight tendency towards a reduction compared to Proteobacteria, which may also lower the content of Fe-S proteins, and thus the supply of Fe-S clusters in these microorganisms. However, the conjecture that this trend constitutes a strategy to reduce the demand and content of Fe-S centers, and thus the physiologically detrimental effects of their oxidation, requires further study.

### Prediction of Systems for the Biosynthesis of Fe-S Clusters in Acidophiles

As described previously, the Isc system corresponds to the housekeeping mechanism for Fe-S center biosynthesis in *E. coli* [9]. Conversely, Suf is an inducible system that is activated when cells are exposed to oxidative stress or iron deprivation [8]. Since acidophiles are highly resistant to oxidative conditions [20, 31], the Suf system could play a relevant role in counteracting the effects of such harsh environmental conditions. Thus, in order to evaluate whether there exists a preferential usage of either of these systems, we inspected the genomic sequences and searched for the presence of *suf* and *isc* genes.

**Fig. 1:**
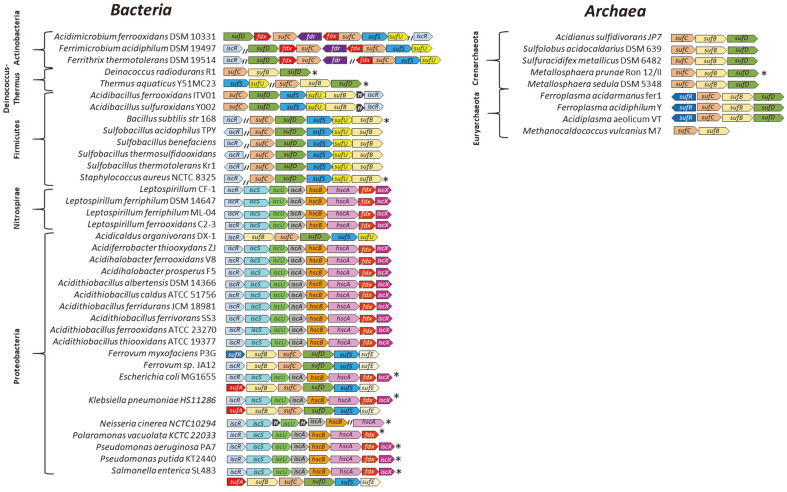
Organization of *suf* and *isc* gene clusters in different microorganisms. Genes are represented by arrows orientated according to the strand location. “*H*”: hypothetical gene, “*fdx*”: ferredoxin and “*fdr*”: ferredoxin reductase. Asterisk (*) indicates neutrophilic microorganisms.

*In-silico* analysis of Fe-S cluster biosynthesis genes in different microorganisms disclosed that acidophilic archaea possess genes encoding for the Suf system only, mostly composed of *sufBCD* genes (Fig. 1), lacking cysteine desulfurase (*sufS* gene). On the other hand, acidophilic bacteria encode complete sets of genes encoding either for a single Suf or an Isc system. The analysis predicts that acidophilic Actinobacteria and Firmicutes synthesize Fe-S clusters by a Suf-like pathway coded by *sufBCDSU* genes, probably regulated by IscR. This pathway has been proposed to be a chimera comprising of homologues of *E. coli* Suf components, and IscU which has been reported as being widely distributed in these lineages [32]. On the other hand, in acidophilic Proteobacteria, *iscRSUAX-hscBA-fdx* appears to be the most represented biosynthesis pathway, since it was detected in *Acidiferrobacter thiooxydans* ZJ, *Acidihalobacter ferrooxidans* V8, *Acidihalobacter prosperus* F5, *Acidithiobacillus albertensis *DSM 14366, *Acidithiobacillus caldus* ATCC51756, *Acidithiobacillus ferridurans* JCM 18981, *Acidithiobacillus ferrivorans* SS3, *Acidithiobacillus ferrooxidans* ATCC 23270 and *Acidithiobacillus thiooxidans* ATCC19377. The presence of the Suf system seems to be an exception among Proteobacteria, since *sufBCDSU-iscR* genes were detected only in the genome of *Acidicaldus organivorans* C2-3. Interestingly, the Nitrospirae phylum harbours the whole *iscRSUAX-hscBA-fdx* gene repertoire for biosynthesis of Fe-S centers. As seen in Fig. 1, unlike the genomes from *E. coli*, *S. enterica* and *K. pneumoniae* that contain the complete set of genes for both the Isc and Suf systems, none of the genomes from the 25 acidophilic bacteria and 8 archaea analysed here showed the simultaneous presence of genes for both systems. In addition, as previously reported extremophilic *D. radiodurans* and *T. aquaticus* genomes encode the Suf system only [18, 33].

**Fig. 2:**
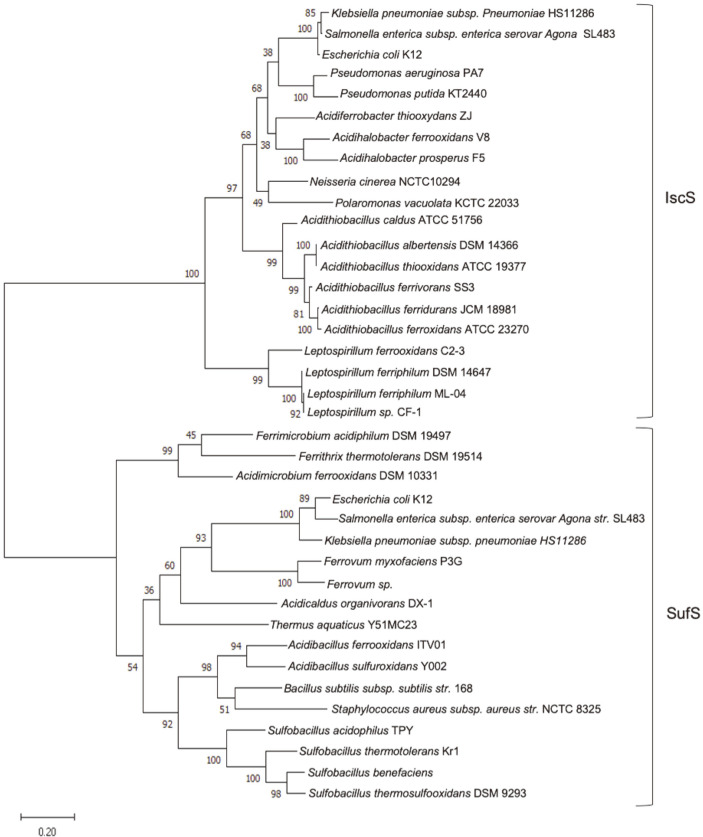
Phylogenetic analysis of SufS and IscS from extremophilic and neutrophilic bacteria. The evolutionary history was inferred by the Maximum Likelihood algorithm and the JTT matrix-based model using amino acidic sequences of SufS and IscS. The analysis was performed using the MEGA-X program.

Interestingly, the phylogenetic analysis (Fig. 2) revealed that IscS and SufS proteins, wich have major roles in providing sulfur for Fe-S cluster synthesis, from acidophilic microorganisms diverge evolutionarily from each other into two defined groups. Additionally, the Isc clade of the Nitrospirae phylum clearly diverged from a clade of neutrophilic/acidophilic Proteobacteria, suggesting that acidic conditions do not represent a significant environmental force in determining the evolution of IscS/SufS desulfurase systems and the corresponding acquisition of new structural features.

### Co-Transcriptional Analysis of the *isc* Gene Cluster from *Leptospirillum* sp. CF-1

Acidophilic microorganisms are considered highly tolerant to the oxidative conditions that prevail in acidic bioleaching environments. Thus, the presence of a unique Isc system in acidophiles that are representative of the Nitrospira and Proteobacteria phyla suggests that, unlike *E. coli*, in these microorganisms this system might be active under oxidative conditions. For this reason, we characterized the transcriptional organization of the genetic cluster *iscRSUAX-hscBA-fdx* that accounts for the *isc* genes in *Leptospirillum* sp. CF-1, a bacterium from Nitrospira phylum that serves as a model microorganism to elucidate the mechanisms used by acidophiles to thrive under highly oxidative conditions [34, 35].

**Fig. 3:**
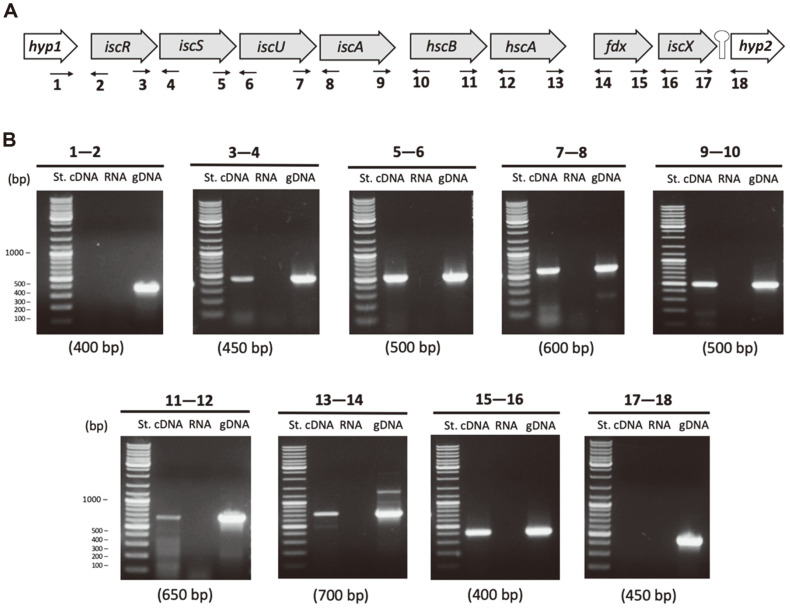
Genetic organization of the *isc* gene cluster in *Leptospirillum* sp. CF-1. (**A**) Schematic map of the *isc* operon. The Rho-independent-like structure between *icsX* and *hyp2* was predicted with the RNAold finding terminator tool. The primers designed to amplify each intergenic region are indicated below the operon (1-18). (**B**) RT-PCR amplification of intergenic regions using cDNA, RNA (without addition of reverse transcriptase) as negative control, and genomic DNA (gDNA) as positive control of amplification. The primer pairs used for amplification are indicated above each panel and the expected amplicon sizes are shown below each panel. St – molecular weight standards.

The eight candidate genes were analyzed to evaluate their co-transcription using a RT-PCR approach in cells grown with ferrous iron under standard growth conditions. As can be seen in Fig. 3, RT-PCR experiments clearly showed that *iscRSUA-hscBA-fdx-iscX* are co-transcribed, demonstrating that these 8 genes form part of a single transcription unit, the Isc operon, in *Leptospirillum* sp. CF-1. The physical arrangement of this cluster of genes, including the flanking non-cotranscribed genes (*hyp1* and *hyp2*) is shown in Fig. 3. In agreement with these results, a rho-independent transcription terminator was predicted bioinformatically. The co-transcription of these eight genes confirms that they are functionally related and similarly activated in response to common physiological signals.

### The Isc System of *Leptospirillum* sp. CF-1 is Up-Regulated under Oxidative Stress and Iron Starvation Conditions

To elucidate whether the Isc operon from *Leptospirillum* sp. CF-1 is active and upregulated under stress conditions, mRNA levels of the gene encoding cysteine desulfurase (IscS) were measured using cells exposed to oxidative conditions (260 mM ferric ion (Fe_2_(SO_4_)_3_)) or to iron starvation by reducing the concentration of ferrous ion either to 50% of the concentration of the culture medium (33 mM), or by eliminating this nutrient for 3 h (0 mM). ROS overproduction and oxidative stress induction by exposure to ferric iron in *Leptospirillum* sp. was previously assessed [25].

**Fig. 4:**
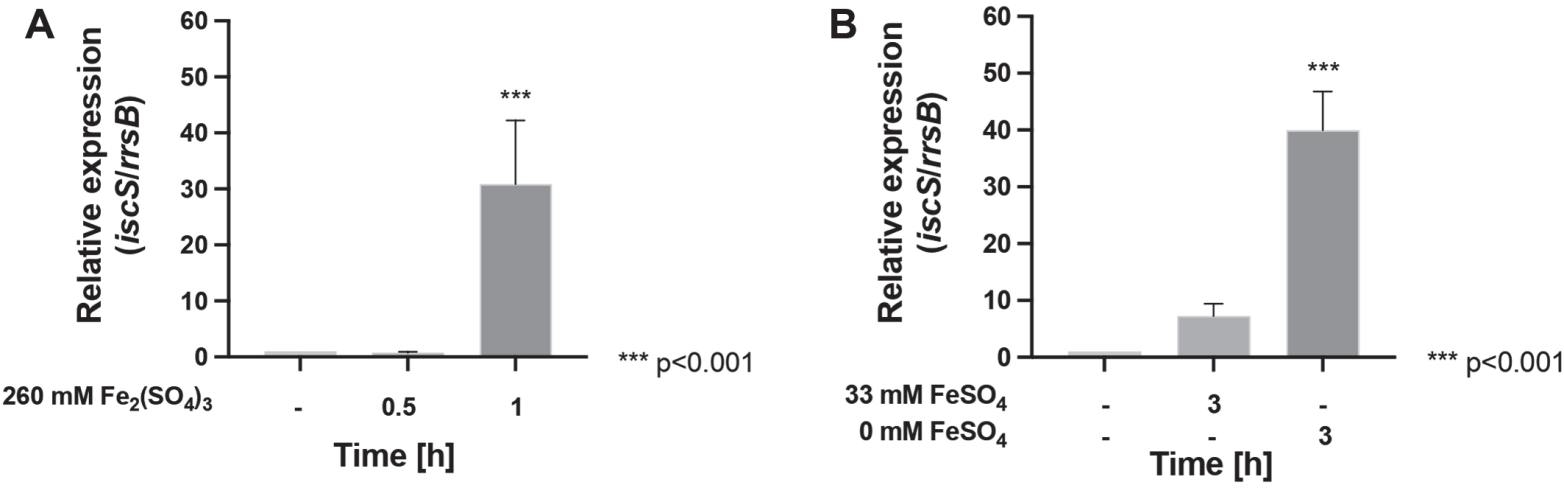
Relative mRNA levels of cysteine desulfurase *iscS* in *Leptospirillum* sp. CF-1. Relative expression was evaluated in cells exposed to (**A**) 260 mM Fe_2_(SO_4_)_3_ for 0.5 and 1 h, and (**B**) decreasing FeSO_4_ concentrations (33 and 0 mM) for 3 h. The data represent the average of 3 independent experiments (bars indicate standard deviation). Statistical analysis was carried out by the ANOVA test.

As observed in Fig. 4, the mRNA levels of *iscS* gene do not show differences in their expression after 0.5 h under oxidative stress conditions with Fe^3+^, however *iscS* expression significantly increased 31-fold (*p* < 0.001) after 1 h of exposure compared to control of non-stressed cells. Concerning iron starvation, the results show that the reduction of Fe^2+^ substrate to 33 mM led to a 7-fold increase in the mRNA level of the *iscS* gene, while the complete depletion of Fe^2+^ significantly-increased expression levels of *iscS* by 40-fold (*p* < 0.001). Altogether, the results suggest that this bacterium actively responds to oxidative stress and iron starvation conditions through an increase of mRNA levels of *isc* genes, thus indicating an important role of this system under stress conditions.

### The Cysteine Desulfurase Activity is Up-Regulated by Oxidative and Iron-Starvation Conditions

**Fig. 5:**
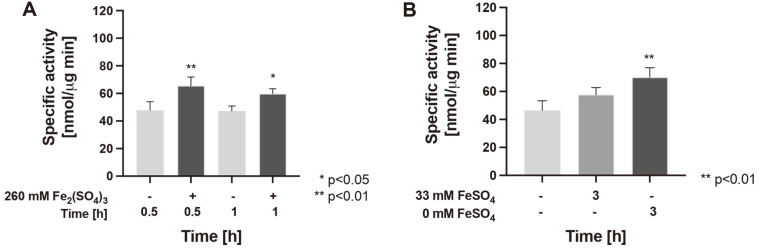
Cysteine desulfurase activity in *Leptospirillum* sp. CF-1. Cysteine desulfurase activity in whole protein extracts from cells exposed to (**A**) 260 mM Fe_2_(SO_4_)_3_ for 0.5 and 1 h, and (**B**) decreasing FeSO_4_ concentrations (33 and 0 mM) for 3 h. The data represent the average of 3 independent experiments (bars indicate the value range). Statistical analysis was carried out by the ANOVA test.

Since mRNA levels of *iscS* showed an increase upon stress induction, cysteine desulfurase (IscS) activity was measured in total cellular extracts derived from cells exposed to oxidative stress and iron starvation conditions. Of note is that genes for other types of desulfurase [36] more than IscS were not detected in the genome of *Leptospirillum* sp. CF-1; therefore the desulfurase activity here detected was more likely due solely to IscS protein. As shown in Fig. 5, the exposure of strain CF-1 to oxidative stress conditions with 260 mM Fe_2_(SO_4_)_3_ led to a significant increase in IscS activity after 0.5 h (137%, *p* < 0.01) and 1 h (126%, *p* < 0.05) compared to the respective non-exposed control cells (100%). Additionally, exposure of the cells to iron starvation for 3 hours increased cysteine desulfurase activity by 124%, while total elimination of this iron source from the medium led to an increase of 151% (*p* < 0.01). Although this increase does not directly correlate with the detected increase in mRNA levels, these results strongly suggest that activity of IscS enzyme from *Leptospirillum* sp. CF-1 is up-regulated as a way of responding to environmental stress conditions.

## Discussion

Fe-S cluster proteins are one of the main targets of ROS. As mentioned above, lowering the content of Fe-S proteins in *D. radiodurans* has been described as a strategy to tolerate highly oxidative conditions [37]. Similarly, recent studies have shown that Fe-S protein levels differ between aerobically and anaerobically cultured cells [9, 38] . Furthermore, it has been suggested that aerobic bacteria preferentially use Fe_2_-S_2_ clusters rather than Fe_4_-S_4_ clusters due to their higher stability under oxidizing environmental conditions [38]. In this respect, the redox status of the environment seems to exert a significant role in determining the content and characteristics of Fe-S proteins in microorganisms. Here, a slight tendency to a lower content of Fe-S proteins in acidophilic compared to neutrophilic proteobacteria was detected, which could represent a partial strategy to face the oxidizing conditions to which the former are permanently exposed. However, in acidophiles there is still a high content of Fe-S proteins, suggesting a high demand for such centers, and the likely existence of mechanisms for protection or repair of Fe-S proteins.

In agreement with previous studies carried out in (hyperthermo)acidophilic archaea, like *Ferroplasma* and *Sulfolobus* [39, 40] and other archaeal representatives [1, 41], the genomic identification of *isc* and *suf* genes revealed that acidophilic archaea possess only genes encoding for the Suf system, where SufCBD seems to represent the core system for Fe-S cluster biosynthesis. Interestingly, homologs of the mandatory cysteine desulfurase (SufS) were not found in any of the genomes analyzed of acidophilic archaea. Also *sufS* genes encoding this enzyme have not been detected in some members of the Archaea domain [42]. As previously suggested, it is possible that sulfides supply sulfur for Fe-S cluster biosynthesis in these archaea [41]. However, a non-canonical desulfurase for liberating S from cysteine or other sources may also exist.

In addition, in this study we found that acidophilic Gram-positive (including Firmicutes and Actinobacteria phyla), and Gram-negative bacteria (including Proteobacteria and Nitrospirae phyla) contain either Suf or Isc biosynthesis pathways, respectively. Surprisingly, we found that only neutrophilic Proteobacteria harbour genes for both Suf and Isc systems. It has been reported that disruption of either the *isc* or the *suf* system does not cause any defects in *E. coli*; however the loss of both pathways causes lethality [43]. Moreover, it has been described that Isc and Suf systems are largely interchangeable, especially in anaerobic environments [43]. As mentioned above, under standard growth conditions, the Isc system is responsible for providing the Fe-S centers of a wide range of proteins, which is consistent with its housekeeping character. Proteins from the Isc system are, in general, more abundant than their Suf counterparts. For its part, the Suf biosynthesis system is more restricted in its variety of substrates, and thus its activity seems to satisfy only the minimum requirements of Fe-S clusters for cell growth. The Suf proteins are more abundant and active under conditions of oxidative stress, due to the higher stability of some of their components under oxidizing conditions [12]. Similarly, this system seems to be more efficient in obtaining iron, and therefore its activity is relevant in iron-deficient conditions. In summary, although the Isc and Suf systems seem to present some functional redundancy, the occurrence of both operons could contribute significantly to the fitness of microorganisms under standard or stress conditions, respectively. However, since most microorganisms harbour just one of the systems, it is possible that differences exist in the stability/activity of Isc or Suf proteins, or in the regulatory networks controlling *isc/suf* gene expression between different microorganisms. To the best of our knowledge, these issues have yet not been addressed in depth.

*Leptospirillum* sp. CF-1, a representative from the Nitrospira class, contains a complete set of *iscRSUA-hscBA-fdx-iscX* genes that were transcribed as a single unit conforming the *isc* operon. Furthermore, strain CF-1 exposed to oxidative stress with ferric ion (Fe^3+^) and iron starvation (Fe^2+^ restriction) exhibited significantly enhanced mRNA levels of the cysteine desulfurase (*iscS*) gene and an increase of cysteine desulfurase (IscS) activity in vitro. In this case, the correlation between mRNA and protein activity levels was low. This fact has been widely reported in different cell types, and may be due to multiple factors such as a low expression of the IscS protein or a decrease in its activity under the physiological conditions assayed, among other factors [44]. In addition, the results reveal interesting information, since unlike that described in *E. coli*, the *isc* operon from *Leptospirillum* CF-1 was activated under stress, suggesting significant differences in underlying regulatory mechanisms that control *isc* gene expression. Regulation of Fe-S cluster assembly pathways in *E. coli* is mainly controlled at the transcriptional level by the repressor IscR [16], a transcription factor containing a Fe-S cluster. This cluster can interconvert from [2Fe-2S] to [2Fe-2S]^1+^ in a stable manner in response to oxidizing or reducing conditions, respectively [45]. Holo-IscR is able to repress transcription of the *iscRSUA* promotor when there is sufficient cluster assembly capacity. Nevertheless, conditions that increase demand for Fe-S clusters (*e.g.*, aerobic growth) are also predicted to increase the derepression of the *isc* operon [46]. For its part, *suf* transcription is kept low due to repression by Fe^2+^-Fur, which binds to the *suf* promoter [10]. During oxidative stress or iron starvation, apo-IscR predominates inside the cell and relieves iscRSUA repression. In addition, apo-IscR activates *suf* transcription in response to oxidative stress as Fur repression is lifted [11]. IscR, along with the OxyR regulator and the integration host factor (IHF), activate the Suf operon under stress conditions, through the binding to specific sites on the *suf* promoter [11]. Finally, *E. coli* exposed to iron starvation conditions have increased expression of the small RNA RyhB, which leads to post-transcriptional repression of the Isc system so that Suf becomes the predominant pathway in this bacterium [16]. Here, we showed that exposure of the strain CF-1 to stress conditions increases the *iscS* transcript level and IscS enzymatic activity. Most likely, IscR is the master regulator of *isc* genes in *Leptospirillum*. Nevertheless, the participation of OxyR in *isc* regulation is unlikely since this regulator has not been detected in members of the genus *Leptospirillum* using bioinformatic approaches [31]. Similarly, a gene for a classic Fur regulator has not been detected in studies of the *Leptospirillum* genome [47]. In this way, the mechanisms involved in IscR-mediated regulation could differ from the classical regulatory system described in *E. coli*. Whether IscR acts as transcriptional depressor or activator of the *isc* operon in *Leptospirillum* remains to be determined.

In summary, this study showed the distribution and the sole presence of either Isc or Suf machinery in acidophilic microorganisms. Data also showed that IscS activity from *Leptospirillum* CF-1 is upregulated upon oxidative stress and iron starvation, suggesting a higher stability and functionality of Isc proteins than their counterparts in *E. coli* under stress conditions. These findings highlight the relevance of the Isc system in facing oxidative conditions in microorganisms that inhabit extremely acidic and metal loaded environments.

## Supplemental Materials

Supplementary data for this paper are available on-line only at http://jmb.or.kr.

## Figures and Tables

**Table 1:** Primers used in this research.

	Number	Gene	Sequence	Tm °C	Amplicon (bp)
RT-PCR	1	*hyp1*	(F) TTCGGGTGTTCTGGGAGCAC	57	400
	2	*iscR*	(R) CGTTTCTGGCCGGTTTCTTC		
	3	*iscR*	(F) TCAATGCGATCGATGGGTCC	60	450
	4	*iscS*	(R) TTCCGCCACGCCTTTGATG		
	5	*iscS*	(F) CTCTCCGGCCGTTTCCAATC	60	500
	6	*iscU*	(R) CGCCCGCATCATTGATCTTG		
	7	*iscU*	(F) AATGCGGCGACGTGATGAAG	60	600
	8	*iscA*	(R) TTTGAATCCGCCTCCCATG		
	9	*iscA*	(F) CGGCATCCGGATCCTGATTG	62	500
	10	*hscB*	(R) TGATCACCTGTTCGCCGGAC		
	11	*hscB*	(F) TTTCCCCGGCTCACTTGATG	60	650
	12	*hscA*	(R) AAGATGGCGGTTGCGGAATG		
	13	*hscA*	(F) TCACCGGAATCGAACCCATG	62	700
	14	*fdx*	(R) AGGCCTTCCGCTTCATCCAG		
	15	*fdx*	(F) CATCCTGAAAGCGGCGCTTC	62	400
	16	*iscX*	(R) TGAAGCCGGGTAGCTCCATC		
	17	*iscX*	(F) TGATGGAGCTACCCGGCTTC	57	450
	18	*hyp2*	(R) CCCGAAGAAGCCGTAACGAG		
RT-qPCR	19	*iscR*	(F) TCGTCAACATCCGGGAGGTC	60	177
	20		(R) CGATCGCATTGATGACGTCG		
	21	*iscS*	(F) GAACCGTGCCAATCGACGTC	62	192
	22		(R) TCCCCGGCGTATTGAGTGTC		
	23	*hscB*	(F) TCGAAAACTCCGCCCTGGTG	63	187
	24		(R) TGCTTTCCGGCGACGTCTTC		
	25	*rrsB*	(F) ACGGGTGAGTAGACATGGG	60	105
	26		(R) GGTAGGGTGCAAACGGG		

(F): forward, (R): reverse.
