# Supplementary material for: Insights into Systems for Iron-Sulfur Cluster Biosynthesis in Acidophilic Microorganisms
Source: J Microbiol Biotechnol. 2022 Aug 19;32(9):1110–9. doi: 10.4014/jmb.2206.06045 (PMC9628965; doi:10.4014/jmb.2206.06045)
Supplement: Supplementary file 1 [file jmb-32-9-1110-supple.pdf]

J. Microbiol. Biotechnol.
https://doi.org/10.4014/jmb.2206.06045

Table S1. Comparison of [Fe-S] proteins content between acidophilic and neutrophilic microorganisms

Domain	Phyla	Microorganisms	Number of [Fe-S] protein/total proteins	Percentage	Optimal temperature (°C)	Optimal pH
Archaea	Crenarchaeota	*Acidianus sulfidivorans* JP7	107/2270	4.7%	74	1.1
		Metallosphaera prunae Ron 12/II	129/2310	5.58%	75	2.75
		Metallosphaera sedula DSM 5348	126/2292	5.5%	70	2.0
		Sulfolobus acidocaldarius DSM 639	88/2260	3.89%	70	3.5
		Sulfuracidifex metallicus DSM 6482 = JCM 9184	99/3339	2.96%	65	1.5
	Euryarchaeota	*Acidiplasma aeolicum* VT	69/1989	3.47%	45	1.5
		Ferroplasma acidarmanus fer1	70/1825	3.84%	42	1.35
		Ferroplasma acidiphilum Y	65/1746	3.72%	35.5	1.75
		Methanocaldococcus vulcanius M7	125/1793	7.03%	80	**6.5**
	Actinobacteria	*Acidimicrobium ferrooxidans* DSM 10331	73/1979	3.69%	47.5	2.0
		Ferrimicrobium acidiphilum DSM 19497	82/3267	2.51%	35	2.0
		Ferrithrix thermotolerans DSM 19514	79/2694	2.93%	43	1.8
	Deinococcus-Thermus	*Deinococcus radiodurans* R1	72/2962	2.43%	30 (Brooks and Murray, 1981)	**7.0**
		Thermus aquaticus Y51MC23	75/2538	2.96%	70 (Brumm et al., 2015)	**7.6**
	Firmicutes	*Acidibacillus ferrooxidans* ITV01	91/3397	2.68%	30	2.0
		Acidibacillus sulfuroxidans Y002	91/3059	2.97%	43	1.8
		Sulfobacillus acidophilus TPY	130/3425	3.8%	48	1.8
		Sulfobacillus benefaciens	145/5423	2.67%	38	0.98
		Sulfobacillus thermosulfidooxidans DSM 9293	110/4078	2.7%	51	1.7
		Sulfobacillus thermotolerans Kr1	106/3121	3.4%	40	1.8
		Staphylococcus aureus subsp. aureus NCTC 8325	49/2687	1.82%	37 (Missiakas and Schneewind, 2018)	**6.5***
		Bacillus subtilis subsp. subtilis str. 168	85/4409	1.93%	30*	**7.5**

Bacteria	Nitrospirae	*Leptospirillum* CF-1	80/2460	3.25%	37	1.75
		Leptospirillum ferriphilum DSM 14647	92/2614	3.52%	37	1.8
		Leptospirillum ferriphilum ML-04	86/2213	3.89%	40	1.85
		Leptospirillum ferrooxidans C2-3	99/2348	4.22%	40	1.75
	Proteobacteria	*Acidicaldus organivorans* DX-1	114/3306	3.45%	52.5	2.75
		Acidiferrobacter thiooxydans ZJ	134/3500	3.83%	33.5	1.0
		Acidihalobacter ferrooxidans V8	114/3072	3.71%	30	1.9
		Acidihalobacter prosperus F5	123/3210	3.83%	30	1.9
		Acidithiobacillus albertensis DSM 14366	98/3922	2.5%	30	3.75
		Acidithiobacillus caldus ATCC 51756	101/3087	3.27%	45	2.25
		Acidithiobacillus ferridurans JCM 18981	122/2656	4.59%	29	2.1
		Acidithiobacillus ferrivorans SS3	126/2982	4.23%	22.5	1.9
		Acidithiobacillus ferrooxidans ATCC 23270	116/2871	4.04%	26	2.3
		Acidithiobacillus thiooxidans ATCC 19377	108/3749	2.88%	26	2.0
		Ferrovum myxofaciens P3G	85/3204	2.65%	32	3.0
		Ferrovum sp.	80/2007	3.99%	28	2.5
		Escherichia coli str. K-12 substr. MG1655	154/4242	3.63%	37	**6.75**
		Klebsiella pneumoniae subsp. pneumoniae HS11286	137/5731	2.39%	37*	**7.0**
		Neisseria cinerea NCTC10294	53/1913	2.77%	37*	**6.75**
		Polaromonas vacuolata KCTC 22033	89/3739	2.38%	4 (Irgens et al., 1996)	**7.0**
		Pseudomonas aeruginosa PA7	140/6009	2.3%	37	**7.5**
		Pseudomonas putida KT2440	128/5715	2.24%	30 (Fonseca et al., 2011)	**7.0**
		Salmonella enterica subsp. enterica serovar Agona str. SL483	150/4484	3.4%	37	**7.0**

Data of pH and temperature were collected from https://acidb.cl/ and uniprot.org.
